# Single-cell spatial profiling identifies a mucosal-like epithelial signature in Hidradenitis suppurativa tunnels

**DOI:** 10.1172/jci.insight.200187

**Published:** 2026-06-22

**Authors:** Peter Dimitrion, Jesse Veenstra, Deangelo Ferguson, Ping Wang, Jeffrey Cruz, Tasneem F. Mohammad, Ian Loveless, Aamir Siddiqui, Iltefat H. Hamzavi, Li Zhou, Indra Adrianto, Qing-Sheng Mi

**Affiliations:** 1 Center for Cutaneous Biology and Immunology Research, Department of Dermatology, Henry Ford Health, Detroit, Michigan, USA.; 2 Department of Plastic Surgery, Henry Ford Health, Detroit, Michigan, USA.; 3 Center for Bioinformatics, Department of Public Health Sciences, Henry Ford Health, Detroit, Michigan, USA.; 4 Department of Medicine, Michigan State University, Lansing, Michigan, USA.; 5 Department of Medicine, Henry Ford Health, Detroit, Michigan, USA.; 6 Henry Ford Health + Michigan State University Health Sciences, Detroit, Michigan, USA.

**Keywords:** Dermatology, Immunology, Bioinformatics, Cellular immune response

## Abstract

Tunnels in HS have a transcriptional signature like mucosal epithelia that may contribute to inflammation in HS.

**To the Editor:** Hidradenitis suppurativa (HS) is an inflammatory skin disease where dermal tunnels (keratinized epithelia) form from ruptured follicular abscesses (1). Immunohistochemistry of tunnels shows atypical keratin staining in tunnel epithelium, suggesting inflammation-driven keratinocyte dysregulation. The gene-expression profile of tunnels relative to lesional (L-epi) and non-lesional epidermis (NL-epi) remains poorly characterized.

We applied spatiotemporal enhanced-resolution omics sequencing (STEREOseq) to lesional and non-lesional skin from a single patient with advanced HS, enabling spatial transcriptomic analysis at single-cell resolution (Supplemental Figure 1A and Supplemental Methods; supplemental material available online with this article; https://doi.org/10.1172/jci.insight.200187DS1). The NL-epi sample was taken from an area more than 10 cm away from any HS lesion (1). We histologically identified a tunnel and then performed STEREOseq on the immediate serial section of the tissue block (Supplemental Figure 1, B and C).

We clustered spots using 100 μm bins to balance spatial resolution and sequencing depth, identifying 18 transcriptionally distinct clusters (Figure 1A and Supplemental Figure 1D). Spatial mapping and gene expression identified cluster 14 as tunnel epithelium, cluster 4 as L-epi, and cluster 6 as NL-epi. Gene ontology analysis showed enrichment of skin development, epithelial morphogenesis, and keratinocyte differentiation pathways in HS tunnel and L-epi (Supplemental Figure 2, A and B), while NL-epi was enriched for cellular homeostasis pathways (Supplemental Figure 2C). Enrichment against GTeX showed that tunnel epithelium had a transcriptional profile resembling esophageal and vaginal mucosa, while L-epi and NL-epi displayed a skin-like gene expression signature (Figure 1B).

Pairwise differential gene expression analysis showed distinct gene expression profiles (Supplemental Figure 3A). Genes upregulated in tunnels included canonical mucosal epithelial markers such as *KRT4*, *KRT13*, *MUC1*, *MUC4*, *MAL*, and *SPRR3*. Mucosal genes *CRNN*, *RHCG*, and *SPRR1A* showed higher fold changes in HS tunnel epithelium than in L-epi compared against NL-epi (Supplemental Figure 3B).

A total of 1,214 genes were uniquely upregulated in tunnel epithelium and 345 in L-epi compared with NL-epi epidermis. (Figure 1C). Ingenuity pathway analysis revealed distinct top 5 pathways enriched in tunnel and L-epi unique upregulated genes (Figure 1, D and E).

We found that tunnels showed high expression of antimicrobial peptide *PI3* and low levels of *KRT10* and that tunnels were surrounded by regions of elevated *IGHG1*, suggesting B cell infiltration adjacent to tunnels, in line with recent findings (Figure 1F) (2, 3). Neither the epidermis nor tunnel expressed high levels of inner root sheath or hair markers (*TCHH*, *KRT25*, *KRT85*). *KRT14* was strongest in the tunnel’s basal layer but absent in the NL hair follicle. The lack of outer root sheath (ORS) markers likely reflects incomplete ORS sampling in the tissue section (Figure 1G).

*KRT16* was expressed throughout the tunnel epithelium, consistent with a reactive phenotype. *KRT13*, typically found in esophageal and vaginal mucosa (Supplemental Figure 3C), was restricted to the apical layer of the tunnel, but absent from L-epi, NL-epi, and NL follicles (Figure 1G).

Bulk RNA-sequencing data from HSOmicsDB demonstrated increased *KRT13* expression in lesional tissue, compared with perilesional tissue (Figure 1H). Pairwise comparisons further revealed elevated *KRT13* in lesional compared with perilesional and HC tissue; no difference was found between perilesional and HC tissue (Supplemental Figure 3D).

We performed immunohistochemistry on an independent cohort of HS (*n* = 9) and HC (*n* = 6) patients, evaluating tunnels, L-Epi, and perilesional epidermis (PL-Epi). KRT13 was expressed in the apical aspect of HS tunnels and in the infundibulum of hair follicles within L-Epi, but absent from HC and PL-Epi (Figure 1, I–L, and Supplemental Figure 4, A–C). KRT13 staining in tunnels was comparable to vaginal and esophageal tissue (Supplemental Figure 4, D and E). Previous studies have identified the presence of tendril structures in patients with HS that represent nascent tunnels (4). We found that tendrils also express upregulated KRT13 (Supplemental Figure 4, F and G).

These data suggest tunnels are not simple extensions of inflamed epidermis but undergo dysregulated differentiation during formation. Our data support a model in which HS tunnel formation is driven by aberrant epithelial differentiation, consistent with previous reports (3, 5). HS tunnels may span very large distances, so the presence of KRT13 in tendril structures at the skin surface raises the question as to whether tunnels arise solely from ORS stem cells or if basal epidermal stem cells may also contribute to tunnel formation. KRT13 may have utility in future translational studies as a marker of tunnel-specific epithelium. Further investigation is needed to identify the upstream inflammatory signals responsible, as these may reveal key mechanisms of disease progression and therapeutic targets to prevent tunnel development. It remains unclear whether this epithelial transition is a reactive response or an active driver of the inflammatory niche in HS. As spatial binning in STEREOseq may mix signals from adjacent cells, additional integrated single-cell and spatial analyses are needed to resolve these questions.

## Conflict of interest

JV served on an advisory board for Sensus Healthcare. IHH served as an investigator (grant to institution) for Pfizer Inc, Bayer, Lenicura, Incyte, Estee Lauder, L’Oreal, Unigen, Avita, Arcutis Biotherapeutics, and Ferndale Laboratories, Inc; as advisory board member for AbbVie; and as a consultant to Galderma Laboratories, LP, Incyte, Pfizer, UCB, Boehringer Ingelheim, Beiersdorf, and Clarify Medical.

## Funding support

This work is the result of NIH funding, in whole or in part, and is subject to the NIH Public Access Policy. Through acceptance of this federal funding, the NIH has been given a right to make the work publicly available in PubMed Central.

- NIH grants R01AR078688 to QSM; R01CA284740 for QSM and LZ; R01AR083553, R21AR079089, R33AR076803 to QSM and IA; and UG3AR085895 to QSM, LZ, and IA.
- Henry Ford Immunology Research Program (T71016 to QSM and T71017 to LZ.

## Supplementary Material

Supplemental data

Supporting data values

## Figures and Tables

**Figure 1:**
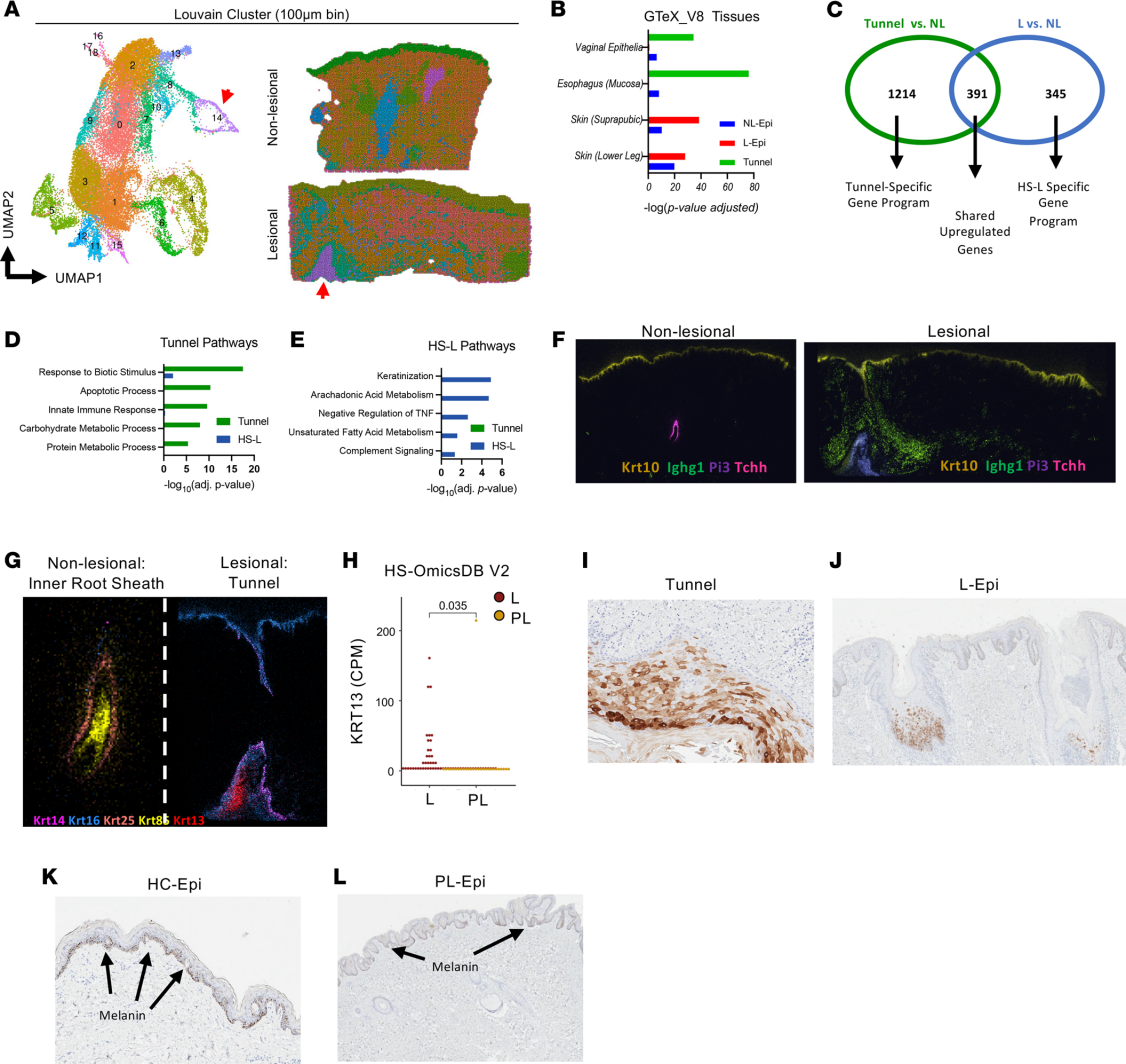
STEREOseq identifies HS tunnels bearing a mucosal epithelia phenotype. (**A**) UMAP plot showing Louvain clustering (100 μm bins; left) and spatial projection (right). Red arrow indicates tunnel cluster. (**B**) Cluster marker genes enriched against GTEx_V8. (**C**) Unique and shared DEGs in tunnel vs. NL-epi and L-epi vs. NL-epi. (**D** and **E**) Top pathways from tunnel- and HS-L–specific genes, respectively. (**F** and **G**) Pseudocolored tissue sections by gene counts (20 μm bins): (**F**) whole tissue and (**G**) tunnel with overlying epidermis. (**H**) KRT13 expression in tunnels from HSOmicsDBv2. (**I**–**L**) KRT13 immunohistochemistry: (**I**) dermal tunnel, (**J**) lesional hair follicle infundibulum, (**K**) HC-epidermis (arrows indicate native melanin pigment), and (**L**) PL-epi. Total magnifcation: ×200 total magnification (**I** and **J**); ×40 total magnification (**K** and **L**). Statistical tests used were hypergeometric test for over-representation analysis (**B**, **D**, and ***E***) and Student’s 2 sided *t* test (**H**).

## Version 1 06/22/2026

Electronic publication
