# Supplementary material for: Single-cell spatial profiling identifies a mucosal-like epithelial signature in Hidradenitis suppurativa tunnels
Source: JCI Insight. 2026 Jun 22;11(12):e200187. doi: 10.1172/jci.insight.200187 (PMC13313476; doi:10.1172/jci.insight.200187)
Supplement: Supplemental data [file jciinsight-11-200187-s167.pdf]

Supplementary Materials and Figures for "Single-Cell Spatial Profiling Identifies a Mucosal-Like Epithelial Signature in Hidradenitis Suppurativa Tunnels"

Dimitrion et. al. 2026

Supplementary Materials and Methods:

Stereo-seq T chip

In this study, we utilized STOmics Stereo-seq T chips (MGI, San Jose, CA) as previously described (Veenstra et al., 2025). The chip surface features DNA nanoballs (DNB) with random barcoded sequences, coordinate identity (CID), molecular identifiers, and polyT sequence-containing oligonucleotides. The DNBs are arranged in a grid-patterned array with spots approximately 220 nm in diameter and 500 nm center-to-center distance. To identify the distinct DNB-CID sequences at each spatial location, single-end sequencing was performed using sequencing primers on a MGIDNBSEQ-Tx sequencer with a SE25 sequencing strategy. CID sequences and their corresponding coordinates for each DNB were determined using a base-calling method according to the manufacturer's instructions for the DNBSEQ sequencer.

Stereo-seq Processing

Tissue samples were sent to MGI, and STEREOseq was performed in their facility under standard manufacturer-recommended procedures. The workflow is outlined briefly here. Pre-frozen OCT tissues were transversely sectioned at 10 μm thickness (Leica CM1950 cyrostat). Tissue sections were adhered to the Stereo-seq chip surface and incubated at 37 °C for 5 minutes. Then, tissues were fixed in methanol and incubated at -20 °C for 30 minutes and then dried. Nuclear staining and imaging were performed with ssDNA Qubit dye and fluorescent microscopy (FITC channel). Immediately adjacent tissue sections were collected for histological examination with hematoxylin and eosin. The tissue on the chip was permeabilized, incubated at 37 °C, and then washed. Released RNA from permeabilized tissues was captured by the DNB and reverse transcribed overnight at 42 °C. After in situ reverse transcription, tissue patches were washed and digested with tissue removal buffer at 55 °C for 10 minutes. cDNA-containing chips were then subjected to cDNA release treatment and collection. The resulting cDNAs were amplified and purified using SPRI-bead

purification for DNB generation. DNB sequencing was performed using a PE100 T7 sequencing kit (50 base R1 for index information + 100 bases R2 for transcript sequence).

Stereo-seq data processing

Fastq files were generated using a DNBSEQ-T7 sequencer, and the raw data were processed according to the Stereo-seq method (https://github.com/STOmics/SAW). CID sequences on the forward reads were first mapped to the designed coordinates of the in situ captured chip, allowing 1 base mismatch to correct for sequencing and PCR errors. Reads with molecular identifiers containing either N bases or more than 2 bases with quality scores lower than 10 were filtered out. After CID mapping and molecular identifier distribution (MID) assignment, retained reads were aligned to the reference genome (Human) using STAR [2]. Mapped reads with MAPQ > 10 were counted and annotated to their corresponding genes. MID with the same CID and the same gene locus were collapsed, allowing 1 mismatch to correct for sequencing and PCR errors. This information was then used to generate a CID-containing expression profile matrix. Stereo-seq data were visualized from the .gef file using Stereomap (https://stereomap.cngb.org/). MIDs were assessed across the Stereo-seq chip to ensure the appropriate signal was localized to tissue regions.

Single-cell segmentation

For cell segmentation, nucleic acid staining images from the same tissue section were projected to the Stereo-seq chip and aligned. We then obtained the X-Y coordinates and overall MID count for each cell using Stereopy (https://github.com/BGIResearch/stereopy) in Python 3.8.18 with a bin size of 100. To remove low-quality cells, the data were filtered to eliminate cells with < 200 genes and >10% mitochondrial genes. After filtering, the remaining 33,527 single cells with 39,145 genes were included in the downstream analyses. The segmented object was converted to a Seurat Object for downstream analysis.

Clustering

Clustering analysis was performed using the Seurat V5 suite of tools. Data was normalized and scaled, and graph-based clustering (Leiden) was performed with a resolution of 0.4. The best-fitting resolution was empirically determined by assessing the biological relevance of clusters generated with known cell populations and regions on the corresponding histology section. We used uniform manifold approximation and projection (UMAP) analysis on the first 28 principal components, which captured 90.5% of the total variance to visualize the distance between cells in 2D space.

DEG Analysis

In Seurat, differentially expressed genes (DEGs) across cell types were identified by comparing the average transcript levels in cells of each cluster to those in all other clusters, using the Wilcoxon Rank Sum test with a bin size of 100. DEGs were identified with a cut-off of an absolute log2 fold change greater than 1, a false discovery rate (FDR) adjusted p-value less than 0.05, and an average count per gene greater than 10. Logistic regression scoring of DEGs in each cluster was determined vs. all other cells, so called "cluster marker genes," which were used for cluster annotation.

Biofunction pathway analysis

GTEx and GO gene lists were compared against identified DEGs using a modified Kolmogorov-Smirnov statistic. Pathways were classified by disease-related biofunctions with a cut-off p-value less than 0.05.

Immunohistochemistry

Formalin-fixed, paraffin-embedded tissue sections (5 μm) were deparaffinized in xylene and rehydrated through a graded ethanol series. Antigen retrieval was performed by incubating sections in citrate buffer (10 mM, pH 6.0) at 95°C for 20 minutes, followed by a gradual cooldown to room temperature. Endogenous peroxidase activity was quenched using 3% hydrogen peroxide in methanol for 10 minutes. To reduce nonspecific background staining, sections were incubated with

Background Sniper (Biocare Medical) for 15 minutes at room temperature. Slides were then incubated overnight at 4°C with mouse monoclonal anti-Cytokeratin 13 antibody (clone 2D7, Thermo Fisher Scientific, MA5-12166) at a 1:10,000 dilution in antibody diluent. Following primary antibody incubation, sections were rinsed and incubated with a species-appropriate biotinylated secondary antibody, followed by avidin-biotin complex (ABC) reagent (Vector Laboratories) for 30 minutes each. Signal was visualized using 3,3′-diaminobenzidine (DAB) as the chromogen, and nuclei were counterstained with hematoxylin. Slides were dehydrated, cleared, and coverslipped with a permanent mounting medium. Negative controls were performed by omitting the primary antibody.

Sex as a biological variable

Our study examined specimens for male and female patients, but we did not compare between male and female specimens in this study.

GTEx Data Utilization

The Genotype-Tissue Expression (GTEx) Project was supported by the Common Fund of the Office of the Director of the National Institutes of Health, and by NCI, NHGRI, NHLBI, NIDA, NIMH, and NINDS. The data used for the analyses described in this manuscript were obtained from: https://gtexportal.org/ on 05/12/24.

Statistics

All statistical analyses were performed using GraphPad Prism (v10) and R (v4.3.1) unless otherwise specified. For comparisons between two groups, an unpaired, two-tailed Student’s t test was used. For comparisons involving more than two groups, one-way or two-way ANOVA with Tukey’s or Sidak’s post hoc correction was applied as appropriate. Nonparametric tests (Mann–Whitney U or Kruskal–Wallis) were used when data did not meet assumptions of normality or equal variance. Correlations were assessed using Pearson’s or Spearman’s rank correlation, depending on data distribution. Differential gene expression analyses were performed using Seurat’s Wilcoxon rank-sum

test with Bonferroni correction for multiple comparisons. Statistical significance was defined as $P < 0.05$. Gene enrichment scores were calculated by comparing the observed expression of each gene within target tissues to its median expression across all reference genesets using a modified z-score approach. Overrepresentation analyses were conducted using the hypergeometric test to identify tissues with significant enrichment of HS-associated transcripts. Adjusted P values were computed using the Benjamini–Hochberg method, with significance defined as FDR < 0.05.

Study Approval

All studies involving human participants were reviewed and approved by the Henry Ford Health Institutional Review Board (Detroit, MI; IRB# 12826). Written informed consent was obtained from all participants prior to enrollment in accordance with the Declaration of Helsinki. For any patient photographs included in this study, written informed consent for publication was obtained, and documentation of consent has been retained by the investigators.

Data Availability

The raw STEREO-seq data generated in this study have been deposited in the Sequence Read Archive (SRA) under accession number SRR36086066 and are publicly available at https://www.ncbi.nlm.nih.gov/sra/?term=SRR36086066. Processed STEREO-seq data have been deposited in Zenodo and are available at https://doi.org/10.5281/zenodo.17617626.

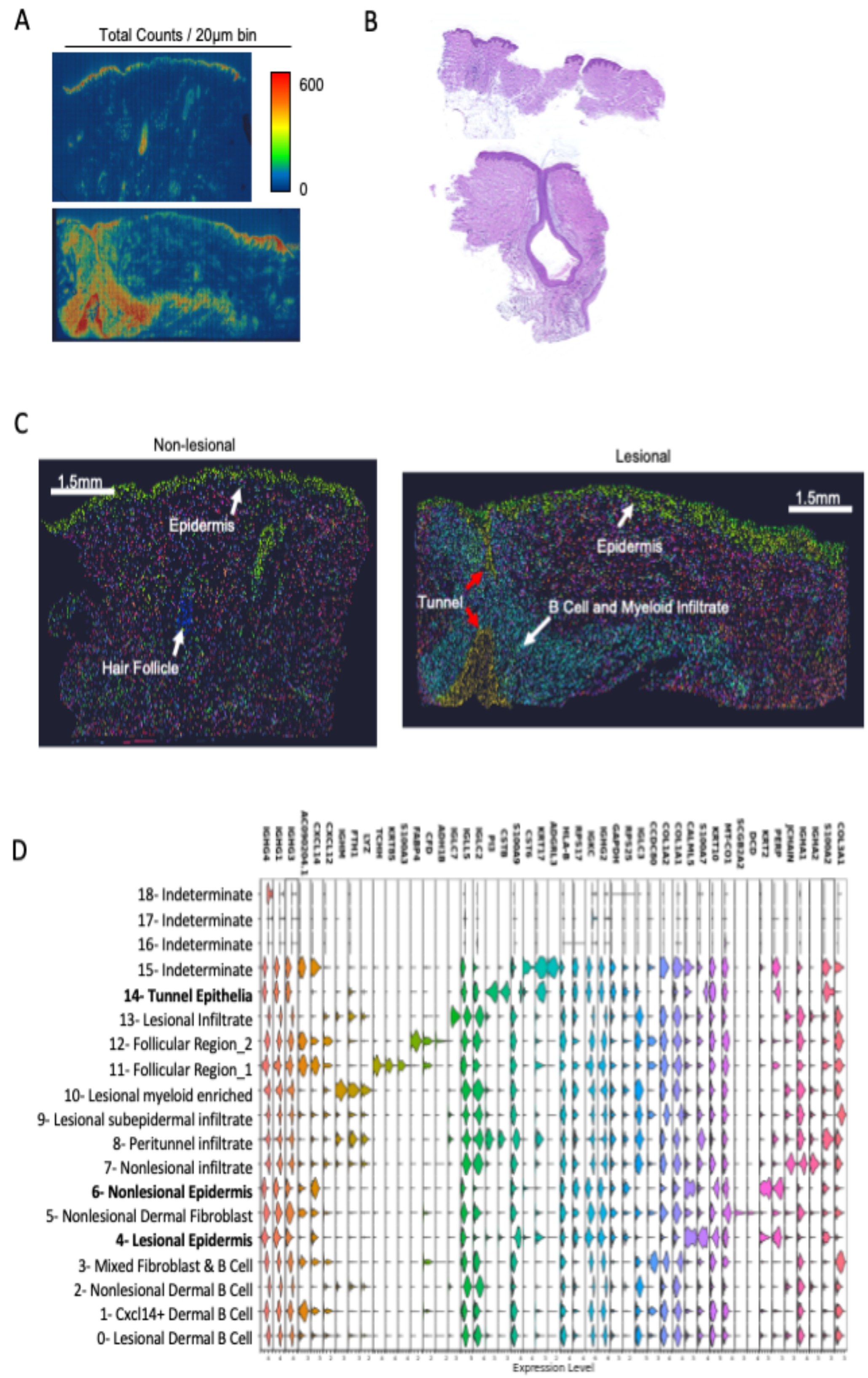

Figure S1: Supporting data for STEREOseq data acquisition and analysis. (A) Spatial heatmap of total counts/20um bin. (B) H&E of immediately prior serial section from a tunnel bearing lesion (lower) and HS-NL skin. (C) Single-cell spatial plot. Cells colored by inferred cell type based on Louvain clustering at 20um bins. (D) Top 2 cluster marker genes for Louvain clusters.

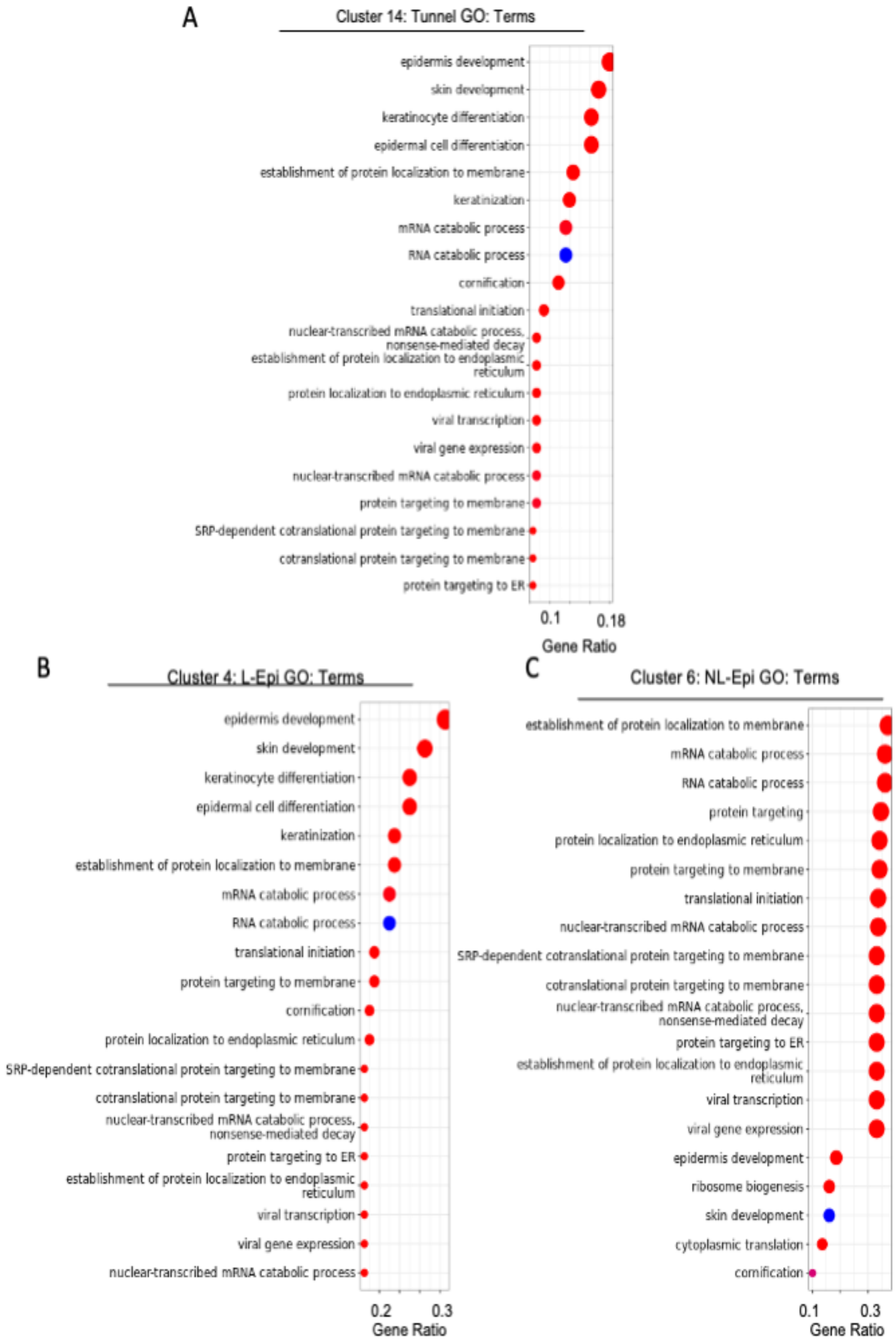

Figure S2: GO Term Enrichment of cluster marker genes for (A) Tunnel; (B) HS-L epidermis; and (C) HS-NL epidermis.

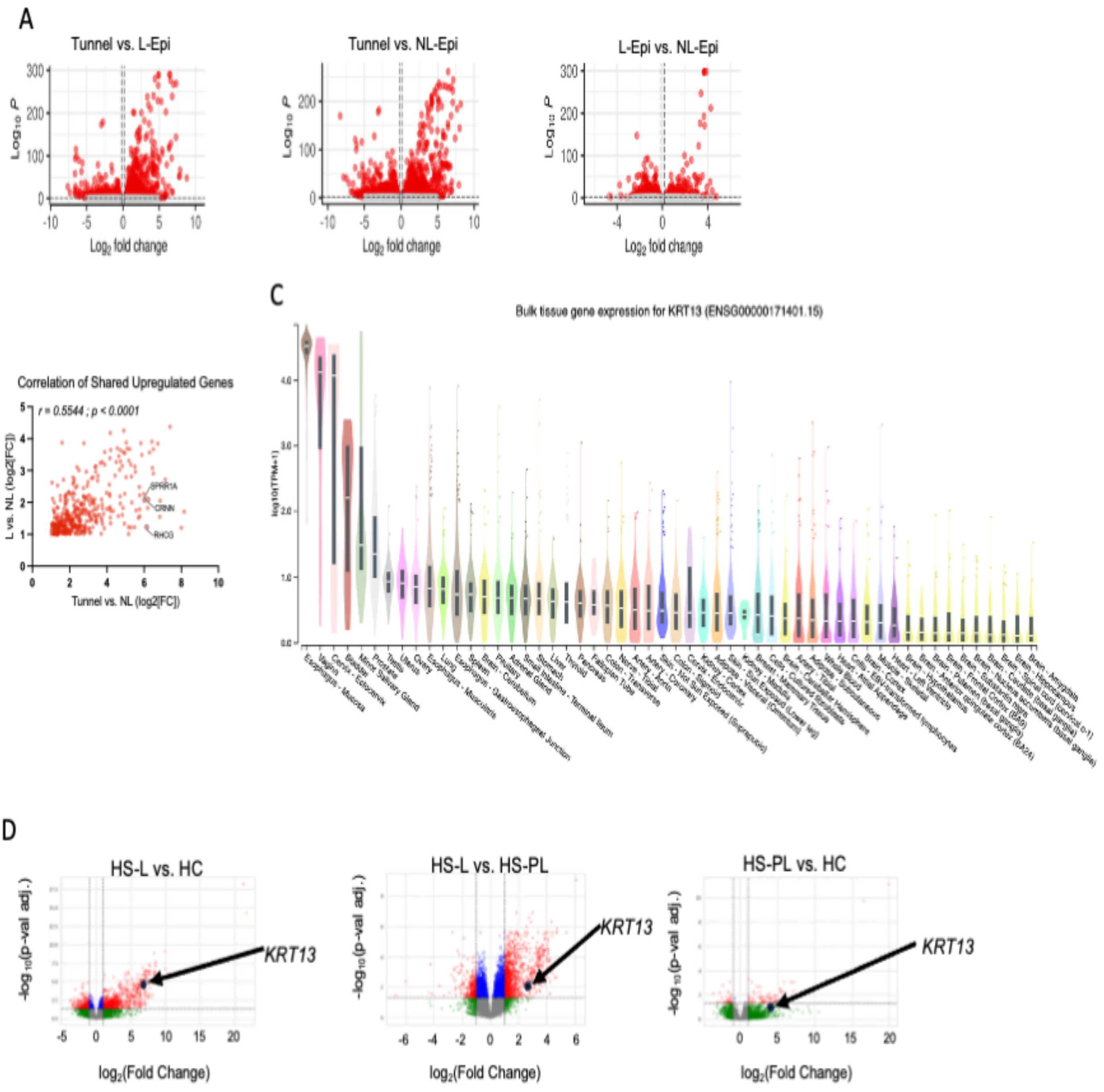

`

Figure S3: Identification of mucosal epithelial genes in tunnel epithelia. (A) DEG analysis from STEREOseq data. (B) Scatter plot of shared upregulated genes between tunnels and HS-L epidermis showing mucosal genes *SPRR1A, CRNN,* and *RHCG* disproportionately elevated in Tunnels. (C) Krt13 expression from GTEx database; (D) Pairwise differential gene expression between HS-L skin (n=73), HS-PL (n=28) and health control (HC) skin (n=19) from HSOmicsDB samples shows elevated KRT13 in lesional HS tissue compared to HS-PL and HC tissue.

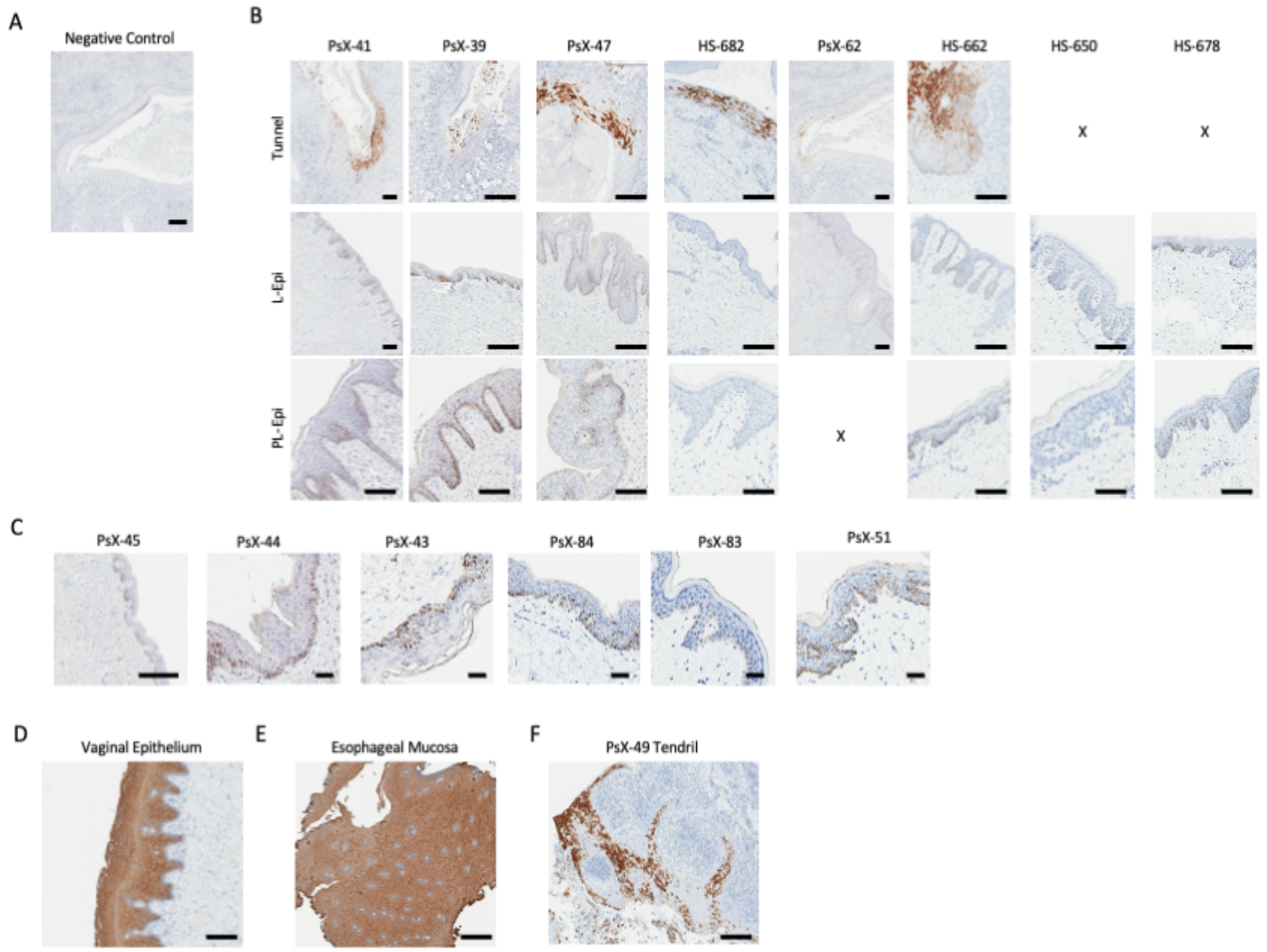

G

Sample	Sex	Ethnicity	Age	Hurley Stage	Location	BMI	Tobacco
PSX39	F	African American	61	III	groins	29.2	Current
PSX41	F	African American	52	III	left axilla	43.86	Former
PSX47	F	African American	24	II&III	bilateral axillary	40	Never
PSX62	M	African American	48	III	buttocks	34	Never
PSX49	F	African American	38	III	buttocks	39.9	Never
HS-682	M	White	32	III	Bilateral axilla	30.43	Never
HS-662	F	African American	35	III	bilateral axilla		
HS650	F	White	24	II	groins	51.72	
HS-678	M	African American	61	III	Bilateral axilla	37.9	Never
PSX43	F	African American	57	Healthy control	abdomen	31	Never
PSX44	F	African American	38	Healthy control	abdomen		Never
PSX45	F	African American	55	Healthy control	abdomen		Never
PSX-83	F	African American	65	Healthy control	abdomen	28.7	No
PSX-84	F	African American	54	Healthy control	abdomen	34.7	No
PSX-51	F	African American	46	Healthy control	abdomen		No

Figure S4: IHC staining for KRT13 of 9 patient samples and 6 HC. (A) IHC negative staining control with secondary antibody only. (B) KRT13 staining on matched tunnel, L-Epi, and PL-Epi from 8 patients. (C) KRT13 staining on HC epidermis. (D) KRT13 staining on vaginal epithelium. (E) KRT13 staining on esophageal mucosa (F) KRT13 staining on HS tendril. (G) Demographics of KRT13 IHC validation cohort.
